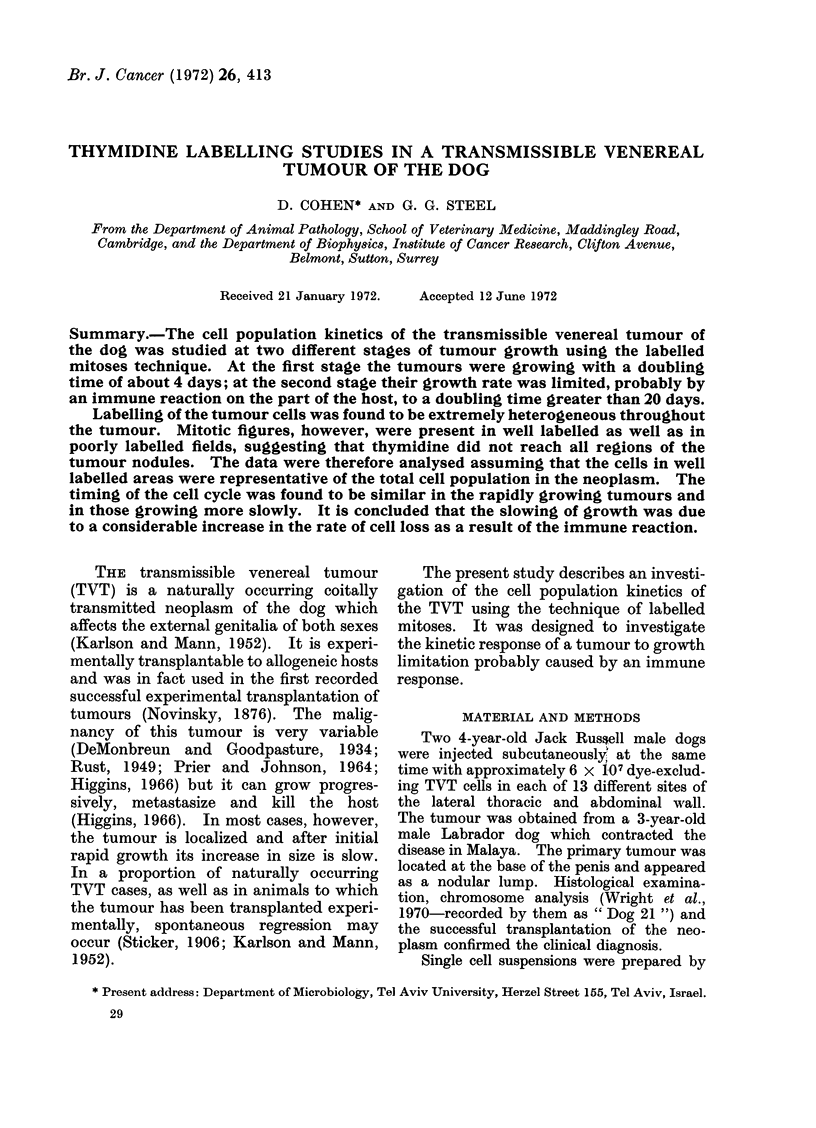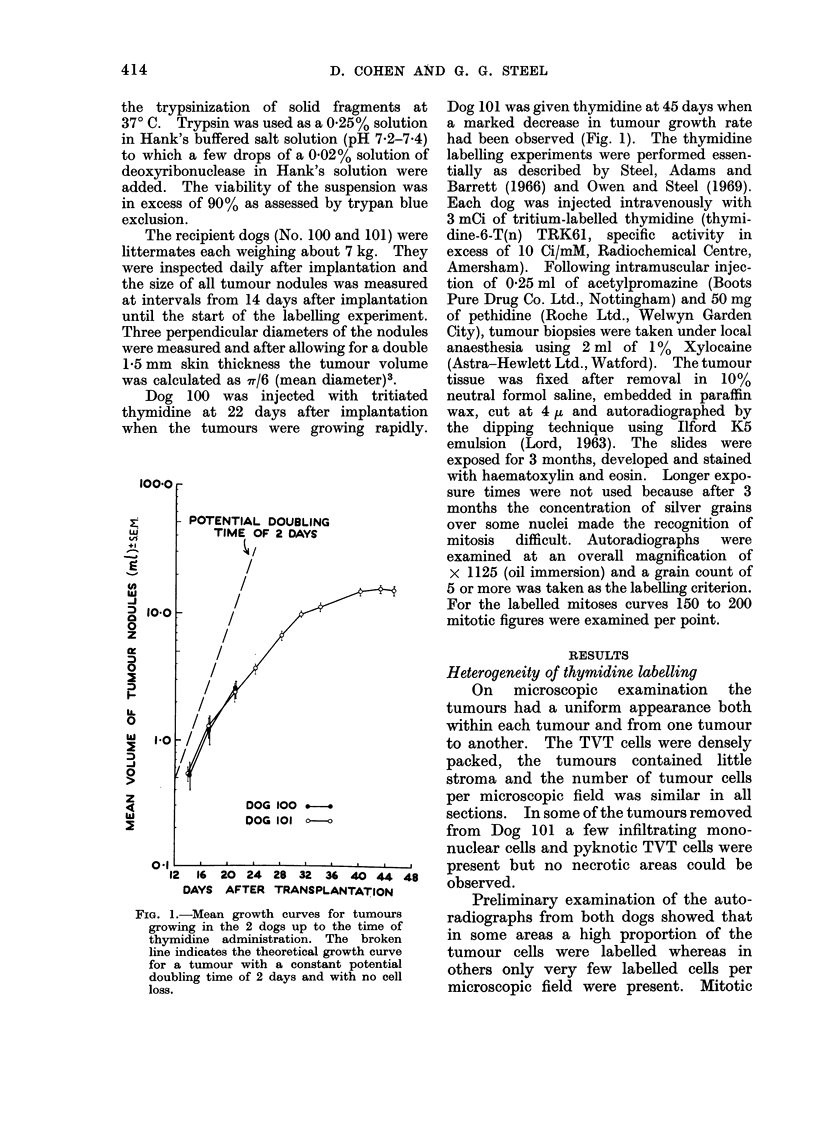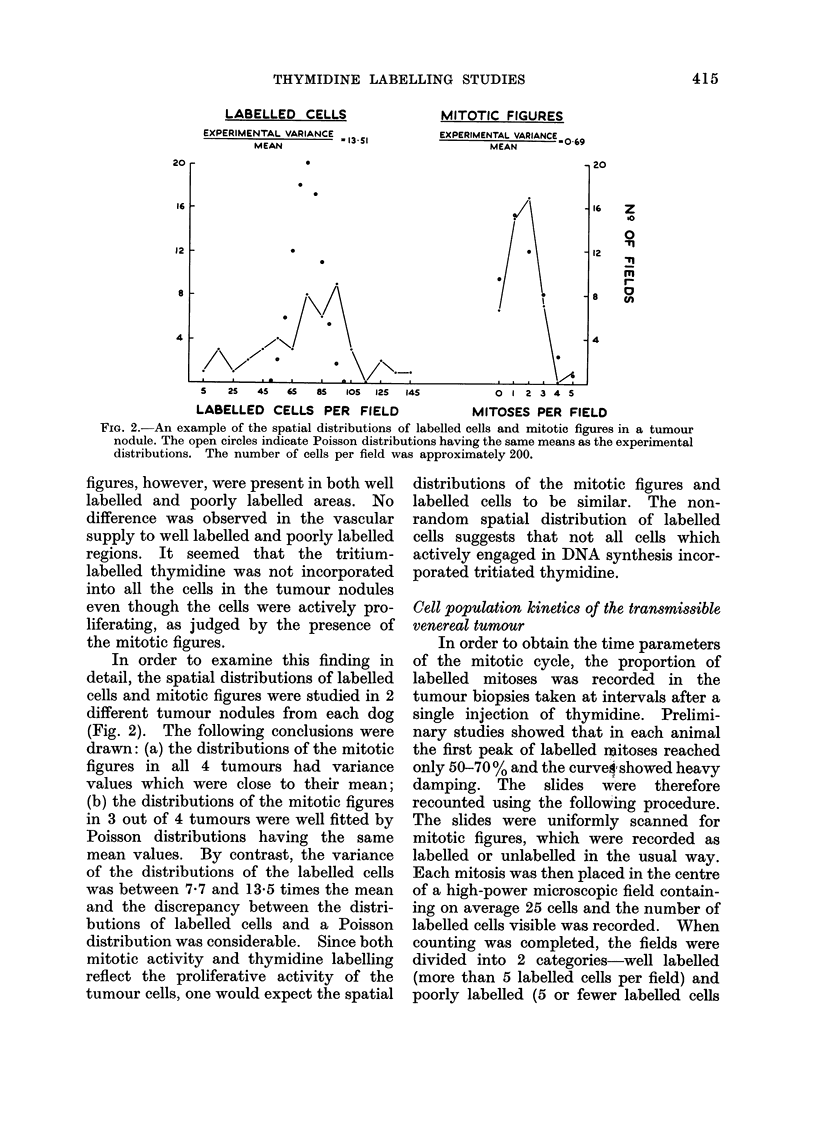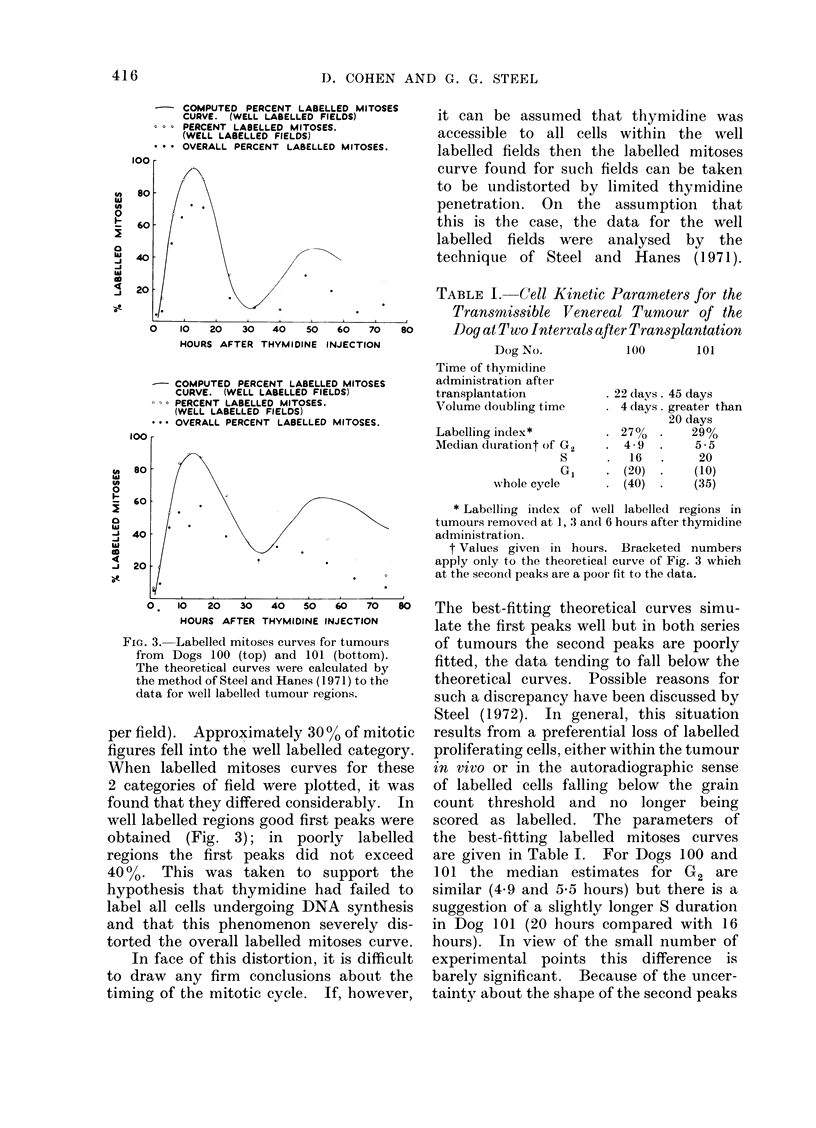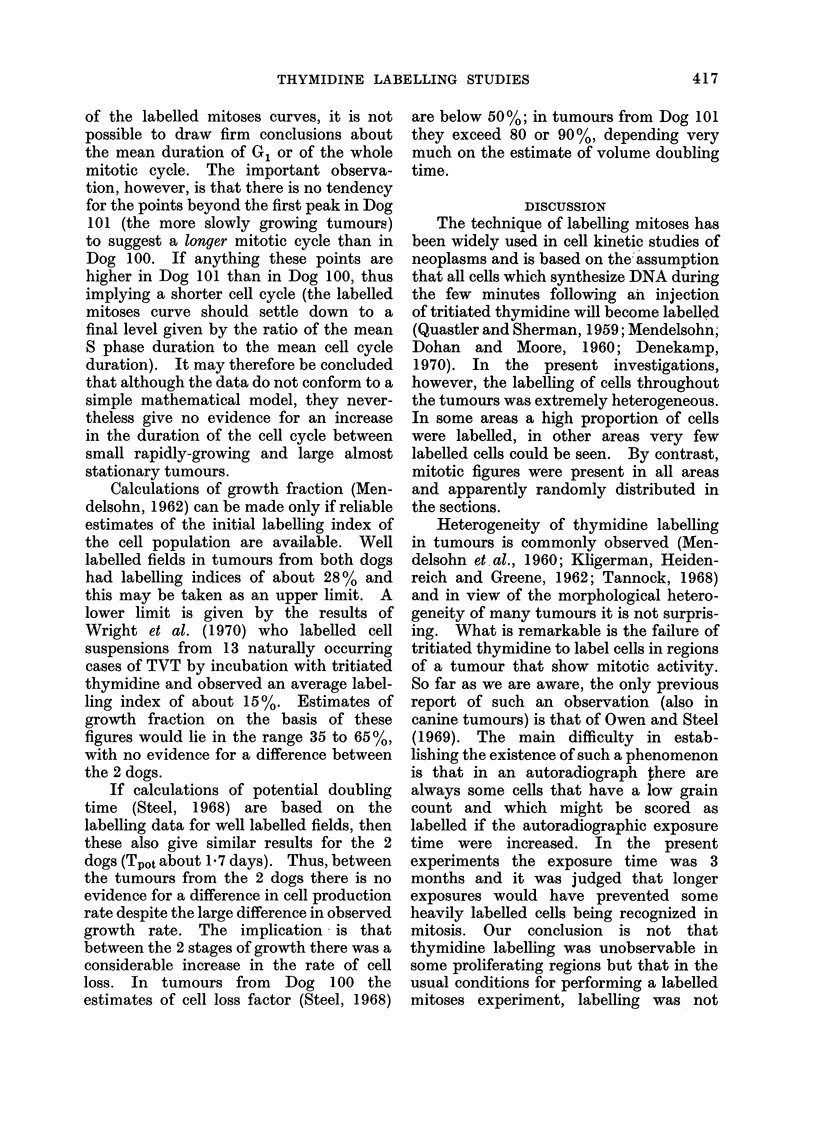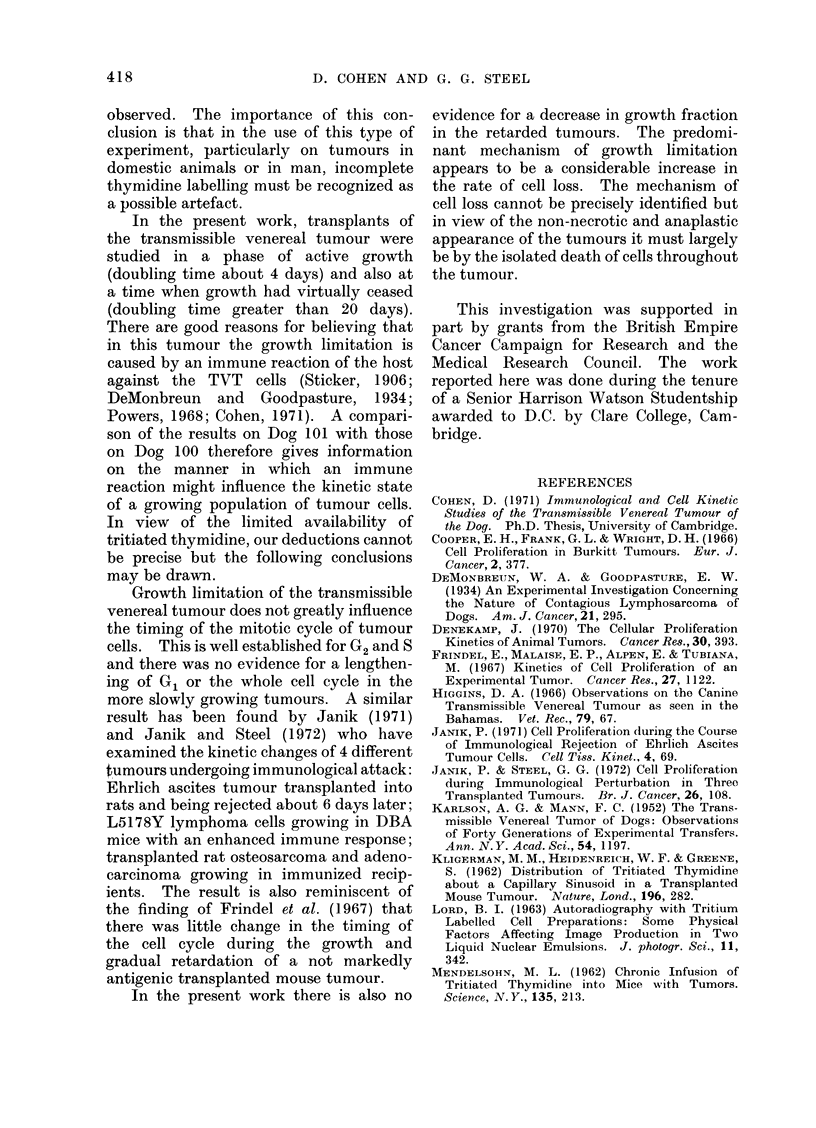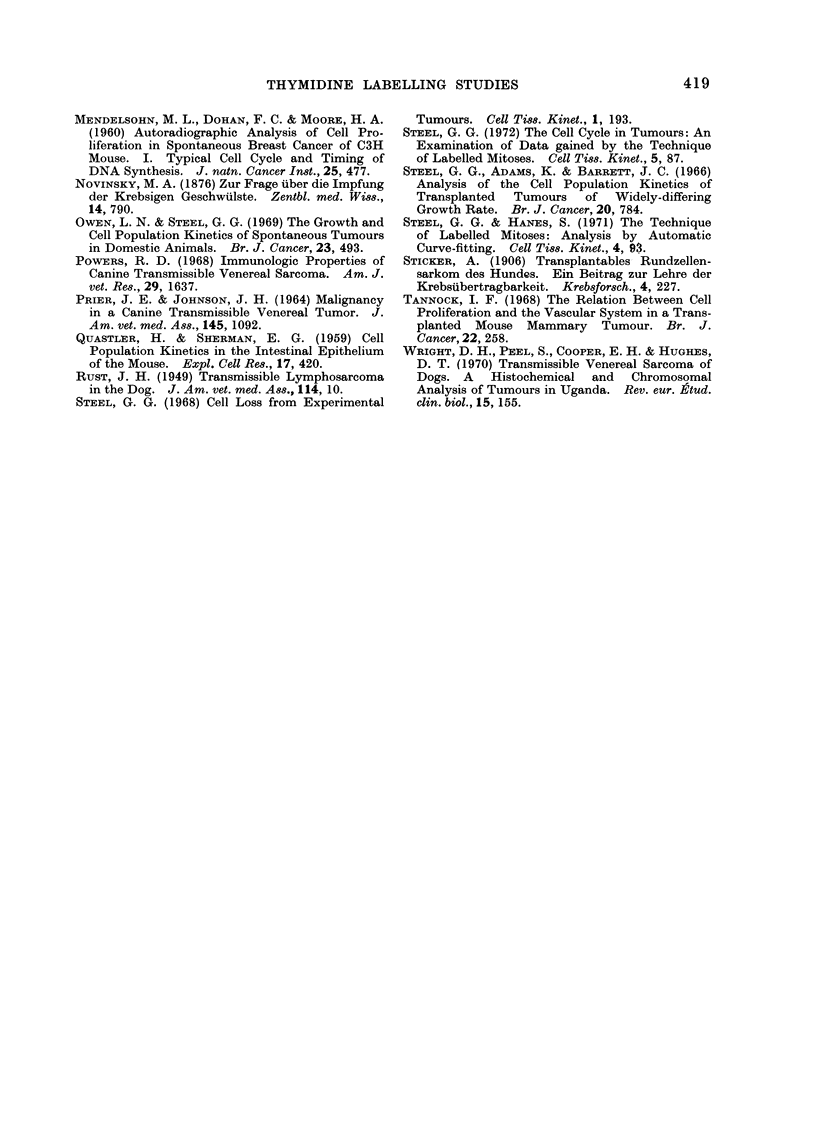# Thymidine Labelling Studies in a Transmissible Venereal Tumour of the Dog

**DOI:** 10.1038/bjc.1972.54

**Published:** 1972-10

**Authors:** D. Cohen, G. G. Steel

## Abstract

The cell population kinetics of the transmissible venereal tumour of the dog was studied at two different stages of tumour growth using the labelled mitoses technique. At the first stage the tumours were growing with a doubling time of about 4 days; at the second stage their growth rate was limited, probably by an immune reaction on the part of the host, to a doubling time greater than 20 days.

Labelling of the tumour cells was found to be extremely heterogeneous throughout the tumour. Mitotic figures, however, were present in well labelled as well as in poorly labelled fields, suggesting that thymidine did not reach all regions of the tumour nodules. The data were therefore analysed assuming that the cells in well labelled areas were representative of the total cell population in the neoplasm. The timing of the cell cycle was found to be similar in the rapidly growing tumours and in those growing more slowly. It is concluded that the slowing of growth was due to a considerable increase in the rate of cell loss as a result of the immune reaction.


*Br. J. Cancer* (1972) **26**, 413

### THYMIDINE LABELLING STUDIES IN A TRANSMISSIBLE VENEREAL TUMOUR OF THE DOG

D. COHEN* AND G. G. STEEL

*From the Department of Animal Pathology, School of Veterinary Medicine, Maddingley Road, Cambridge, and the Department of Biophysics, Institute of Cancer Research, Clifton Avenue, Belmont, Sutton, Surrey*

Received 21 January 1972. Accepted 12 June 1972

**Summary.—The cell population kinetics of the transmissible venereal tumour of the dog was studied at two different stages of tumour growth using the labelled mitoses technique. At the first stage the tumours were growing with a doubling time of about 4 days; at the second stage their growth rate was limited, probably by an immune reaction on the part of the host, to a doubling time greater than 20 days.**

**Labelling of the tumour cells was found to be extremely heterogeneous throughout the tumour. Mitotic figures, however, were present in well labelled as well as in poorly labelled fields, suggesting that thymidine did not reach all regions of the tumour nodules. The data were therefore analysed assuming that the cells in well labelled areas were representative of the total cell population in the neoplasm. The timing of the cell cycle was found to be similar in the rapidly growing tumours and in those growing more slowly. It is concluded that the slowing of growth was due to a considerable increase in the rate of cell loss as a result of the immune reaction.**

THE transmissible venereal tumour (TVT) is a naturally occurring coitally transmitted neoplasm of the dog which affects the external genitalia of both sexes (Karlson and Mann, 1952). It is experimentally transplantable to allogeneic hosts and was in fact used in the first recorded successful experimental transplantation of tumours (Novinsky, 1876). The malignancy of this tumour is very variable (DeMonbreun and Goodpasture, 1934; Rust, 1949; Prier and Johnson, 1964; Higgins, 1966) but it can grow progressively, metastasize and kill the host (Higgins, 1966). In most cases, however, the tumour is localized and after initial rapid growth its increase in size is slow. In a proportion of naturally occurring TVT cases, as well as in animals to which the tumour has been transplanted experimentally, spontaneous regression may occur (Sticker, 1906; Karlson and Mann, 1952).

The present study describes an investigation of the cell population kinetics of the TVT using the technique of labelled mitoses. It was designed to investigate the kinetic response of a tumour to growth limitation probably caused by an immune response.

### MATERIAL AND METHODS

Two 4-year-old Jack Russell male dogs were injected subcutaneously at the same time with approximately $6 \times 10^7$ dye-excluding TVT cells in each of 13 different sites of the lateral thoracic and abdominal wall. The tumour was obtained from a 3-year-old male Labrador dog which contracted the disease in Malaya. The primary tumour was located at the base of the penis and appeared as a nodular lump. Histological examination, chromosome analysis (Wright *et al.*, 1970—recorded by them as " Dog 21 ") and the successful transplantation of the neoplasm confirmed the clinical diagnosis.

Single cell suspensions were prepared by

* Present address: Department of Microbiology, Tel Aviv University, Herzel Street 155, Tel Aviv, Israel.

the trypsinization of solid fragments at 37° C. Trypsin was used as a 0·25% solution in Hank's buffered salt solution (pH 7·2–7·4) to which a few drops of a 0·02% solution of deoxyribonuclease in Hank's solution were added. The viability of the suspension was in excess of 90% as assessed by trypan blue exclusion.

The recipient dogs (No. 100 and 101) were littermates each weighing about 7 kg. They were inspected daily after implantation and the size of all tumour nodules was measured at intervals from 14 days after implantation until the start of the labelling experiment. Three perpendicular diameters of the nodules were measured and after allowing for a double 1·5 mm skin thickness the tumour volume was calculated as $\pi/6$ (mean diameter)$^3$.

Dog 100 was injected with tritiated thymidine at 22 days after implantation when the tumours were growing rapidly.

FIG. 1.—Mean growth curves for tumours growing in the 2 dogs up to the time of thymidine administration. The broken line indicates the theoretical growth curve for a tumour with a constant potential doubling time of 2 days and with no cell loss.

Dog 101 was given thymidine at 45 days when a marked decrease in tumour growth rate had been observed (Fig. 1). The thymidine labelling experiments were performed essentially as described by Steel, Adams and Barrett (1966) and Owen and Steel (1969). Each dog was injected intravenously with 3 mCi of tritium-labelled thymidine (thymidine-6-T(n) TRK61, specific activity in excess of 10 Ci/mM, Radiochemical Centre, Amersham). Following intramuscular injection of 0·25 ml of acetylpromazine (Boots Pure Drug Co. Ltd., Nottingham) and 50 mg of pethidine (Roche Ltd., Welwyn Garden City), tumour biopsies were taken under local anaesthesia using 2 ml of 1% Xylocaine (Astra–Hewlett Ltd., Watford). The tumour tissue was fixed after removal in 10% neutral formol saline, embedded in paraffin wax, cut at 4 $\mu$ and autoradiographed by the dipping technique using Ilford K5 emulsion (Lord, 1963). The slides were exposed for 3 months, developed and stained with haematoxylin and eosin. Longer exposure times were not used because after 3 months the concentration of silver grains over some nuclei made the recognition of mitosis difficult. Autoradiographs were examined at an overall magnification of $\times$ 1125 (oil immersion) and a grain count of 5 or more was taken as the labelling criterion. For the labelled mitoses curves 150 to 200 mitotic figures were examined per point.

### RESULTS

### *Heterogeneity of thymidine labelling*

On microscopic examination the tumours had a uniform appearance both within each tumour and from one tumour to another. The TVT cells were densely packed, the tumours contained little stroma and the number of tumour cells per microscopic field was similar in all sections. In some of the tumours removed from Dog 101 a few infiltrating mononuclear cells and pyknotic TVT cells were present but no necrotic areas could be observed.

Preliminary examination of the autoradiographs from both dogs showed that in some areas a high proportion of the tumour cells were labelled whereas in others only very few labelled cells per microscopic field were present. Mitotic

FIG. 2.—An example of the spatial distributions of labelled cells and mitotic figures in a tumour nodule. The open circles indicate Poisson distributions having the same means as the experimental distributions. The number of cells per field was approximately 200.

figures, however, were present in both well labelled and poorly labelled areas. No difference was observed in the vascular supply to well labelled and poorly labelled regions. It seemed that the tritium-labelled thymidine was not incorporated into all the cells in the tumour nodules even though the cells were actively proliferating, as judged by the presence of the mitotic figures.

In order to examine this finding in detail, the spatial distributions of labelled cells and mitotic figures were studied in 2 different tumour nodules from each dog (Fig. 2). The following conclusions were drawn: (a) the distributions of the mitotic figures in all 4 tumours had variance values which were close to their mean; (b) the distributions of the mitotic figures in 3 out of 4 tumours were well fitted by Poisson distributions having the same mean values. By contrast, the variance of the distributions of the labelled cells was between 7·7 and 13·5 times the mean and the discrepancy between the distributions of labelled cells and a Poisson distribution was considerable. Since both mitotic activity and thymidine labelling reflect the proliferative activity of the tumour cells, one would expect the spatial distributions of the mitotic figures and labelled cells to be similar. The non-random spatial distribution of labelled cells suggests that not all cells which actively engaged in DNA synthesis incorporated tritiated thymidine.

*Cell population kinetics of the transmissible venereal tumour*

In order to obtain the time parameters of the mitotic cycle, the proportion of labelled mitoses was recorded in the tumour biopsies taken at intervals after a single injection of thymidine. Preliminary studies showed that in each animal the first peak of labelled mitoses reached only 50–70 % and the curves showed heavy damping. The slides were therefore recounted using the following procedure. The slides were uniformly scanned for mitotic figures, which were recorded as labelled or unlabelled in the usual way. Each mitosis was then placed in the centre of a high-power microscopic field containing on average 25 cells and the number of labelled cells visible was recorded. When counting was completed, the fields were divided into 2 categories—well labelled (more than 5 labelled cells per field) and poorly labelled (5 or fewer labelled cells

FIG. 3.—Labelled mitoses curves for tumours from Dogs 100 (top) and 101 (bottom). The theoretical curves were calculated by the method of Steel and Hanes (1971) to the data for well labelled tumour regions.

per field). Approximately 30% of mitotic figures fell into the well labelled category. When labelled mitoses curves for these 2 categories of field were plotted, it was found that they differed considerably. In well labelled regions good first peaks were obtained (Fig. 3); in poorly labelled regions the first peaks did not exceed 40%. This was taken to support the hypothesis that thymidine had failed to label all cells undergoing DNA synthesis and that this phenomenon severely distorted the overall labelled mitoses curve.

In face of this distortion, it is difficult to draw any firm conclusions about the timing of the mitotic cycle. If, however, it can be assumed that thymidine was accessible to all cells within the well labelled fields then the labelled mitoses curve found for such fields can be taken to be undistorted by limited thymidine penetration. On the assumption that this is the case, the data for the well labelled fields were analysed by the technique of Steel and Hanes (1971).

TABLE I.—*Cell Kinetic Parameters for the Transmissible Venereal Tumour of the Dog at Two Intervals after Transplantation*

| Dog No. | 100 | 101 |
|---|---|---|
| Time of thymidine administration after transplantation | 22 days | 45 days |
| Volume doubling time | 4 days | greater than 20 days |
| Labelling index* | 27% | 29% |
| Median duration† of $G_2$ | 4·9 | 5·5 |
| S | 16 | 20 |
| $G_1$ | (20) | (10) |
| whole cycle | (40) | (35) |

* Labelling index of well labelled regions in tumours removed at 1, 3 and 6 hours after thymidine administration.

† Values given in hours. Bracketed numbers apply only to the theoretical curve of Fig. 3 which at the second peaks are a poor fit to the data.

The best-fitting theoretical curves simulate the first peaks well but in both series of tumours the second peaks are poorly fitted, the data tending to fall below the theoretical curves. Possible reasons for such a discrepancy have been discussed by Steel (1972). In general, this situation results from a preferential loss of labelled proliferating cells, either within the tumour *in vivo* or in the autoradiographic sense of labelled cells falling below the grain count threshold and no longer being scored as labelled. The parameters of the best-fitting labelled mitoses curves are given in Table I. For Dogs 100 and 101 the median estimates for $G_2$ are similar (4·9 and 5·5 hours) but there is a suggestion of a slightly longer S duration in Dog 101 (20 hours compared with 16 hours). In view of the small number of experimental points this difference is barely significant. Because of the uncertainty about the shape of the second peaks of the labelled mitoses curves, it is not possible to draw firm conclusions about the mean duration of $G_1$ or of the whole mitotic cycle. The important observation, however, is that there is no tendency for the points beyond the first peak in Dog 101 (the more slowly growing tumours) to suggest a *longer* mitotic cycle than in Dog 100. If anything these points are higher in Dog 101 than in Dog 100, thus implying a shorter cell cycle (the labelled mitoses curve should settle down to a final level given by the ratio of the mean S phase duration to the mean cell cycle duration). It may therefore be concluded that although the data do not conform to a simple mathematical model, they nevertheless give no evidence for an increase in the duration of the cell cycle between small rapidly-growing and large almost stationary tumours.

Calculations of growth fraction (Mendelsohn, 1962) can be made only if reliable estimates of the initial labelling index of the cell population are available. Well labelled fields in tumours from both dogs had labelling indices of about 28% and this may be taken as an upper limit. A lower limit is given by the results of Wright *et al.* (1970) who labelled cell suspensions from 13 naturally occurring cases of TVT by incubation with tritiated thymidine and observed an average labelling index of about 15%. Estimates of growth fraction on the basis of these figures would lie in the range 35 to 65%, with no evidence for a difference between the 2 dogs.

If calculations of potential doubling time (Steel, 1968) are based on the labelling data for well labelled fields, then these also give similar results for the 2 dogs ($T_{pot}$ about 1·7 days). Thus, between the tumours from the 2 dogs there is no evidence for a difference in cell production rate despite the large difference in observed growth rate. The implication is that between the 2 stages of growth there was a considerable increase in the rate of cell loss. In tumours from Dog 100 the estimates of cell loss factor (Steel, 1968) are below 50%; in tumours from Dog 101 they exceed 80 or 90%, depending very much on the estimate of volume doubling time.

### DISCUSSION

The technique of labelling mitoses has been widely used in cell kinetic studies of neoplasms and is based on the assumption that all cells which synthesize DNA during the few minutes following an injection of tritiated thymidine will become labelled (Quastler and Sherman, 1959; Mendelsohn, Dohan and Moore, 1960; Denekamp, 1970). In the present investigations, however, the labelling of cells throughout the tumours was extremely heterogeneous. In some areas a high proportion of cells were labelled, in other areas very few labelled cells could be seen. By contrast, mitotic figures were present in all areas and apparently randomly distributed in the sections.

Heterogeneity of thymidine labelling in tumours is commonly observed (Mendelsohn *et al.*, 1960; Kligerman, Heidenreich and Greene, 1962; Tannock, 1968) and in view of the morphological heterogeneity of many tumours it is not surprising. What is remarkable is the failure of tritiated thymidine to label cells in regions of a tumour that show mitotic activity. So far as we are aware, the only previous report of such an observation (also in canine tumours) is that of Owen and Steel (1969). The main difficulty in establishing the existence of such a phenomenon is that in an autoradiograph there are always some cells that have a low grain count and which might be scored as labelled if the autoradiographic exposure time were increased. In the present experiments the exposure time was 3 months and it was judged that longer exposures would have prevented some heavily labelled cells being recognized in mitosis. Our conclusion is not that thymidine labelling was unobservable in some proliferating regions but that in the usual conditions for performing a labelled mitoses experiment, labelling was not observed. The importance of this conclusion is that in the use of this type of experiment, particularly on tumours in domestic animals or in man, incomplete thymidine labelling must be recognized as a possible artefact.

In the present work, transplants of the transmissible venereal tumour were studied in a phase of active growth (doubling time about 4 days) and also at a time when growth had virtually ceased (doubling time greater than 20 days). There are good reasons for believing that in this tumour the growth limitation is caused by an immune reaction of the host against the TVT cells (Sticker, 1906; DeMonbreun and Goodpasture, 1934; Powers, 1968; Cohen, 1971). A comparison of the results on Dog 101 with those on Dog 100 therefore gives information on the manner in which an immune reaction might influence the kinetic state of a growing population of tumour cells. In view of the limited availability of tritiated thymidine, our deductions cannot be precise but the following conclusions may be drawn.

Growth limitation of the transmissible venereal tumour does not greatly influence the timing of the mitotic cycle of tumour cells. This is well established for $G_2$ and S and there was no evidence for a lengthening of $G_1$ or the whole cell cycle in the more slowly growing tumours. A similar result has been found by Janik (1971) and Janik and Steel (1972) who have examined the kinetic changes of 4 different tumours undergoing immunological attack: Ehrlich ascites tumour transplanted into rats and being rejected about 6 days later; L5178Y lymphoma cells growing in DBA mice with an enhanced immune response; transplanted rat osteosarcoma and adenocarcinoma growing in immunized recipients. The result is also reminiscent of the finding of Frindel *et al.* (1967) that there was little change in the timing of the cell cycle during the growth and gradual retardation of a not markedly antigenic transplanted mouse tumour.

In the present work there is also no evidence for a decrease in growth fraction in the retarded tumours. The predominant mechanism of growth limitation appears to be a considerable increase in the rate of cell loss. The mechanism of cell loss cannot be precisely identified but in view of the non-necrotic and anaplastic appearance of the tumours it must largely be by the isolated death of cells throughout the tumour.

This investigation was supported in part by grants from the British Empire Cancer Campaign for Research and the Medical Research Council. The work reported here was done during the tenure of a Senior Harrison Watson Studentship awarded to D.C. by Clare College, Cambridge.

### REFERENCES

COHEN, D. (1971) *Immunological and Cell Kinetic Studies of the Transmissible Venereal Tumour of the Dog.* Ph.D. Thesis, University of Cambridge.

COOPER, E. H., FRANK, G. L. & WRIGHT, D. H. (1966) Cell Proliferation in Burkitt Tumours. *Eur. J. Cancer*, **2**, 377.

DEMONBREUN, W. A. & GOODPASTURE, E. W. (1934) An Experimental Investigation Concerning the Nature of Contagious Lymphosarcoma of Dogs. *Am. J. Cancer*, **21**, 295.

DENEKAMP, J. (1970) The Cellular Proliferation Kinetics of Animal Tumors. *Cancer Res.*, **30**, 393.

FRINDEL, E., MALAISE, E. P., ALPEN, E. & TUBIANA, M. (1967) Kinetics of Cell Proliferation of an Experimental Tumor. *Cancer Res.*, **27**, 1122.

HIGGINS, D. A. (1966) Observations on the Canine Transmissible Venereal Tumour as seen in the Bahamas. *Vet. Rec.*, **79**, 67.

JANIK, P. (1971) Cell Proliferation during the Course of Immunological Rejection of Ehrlich Ascites Tumour Cells. *Cell Tiss. Kinet.*, **4**, 69.

JANIK, P. & STEEL, G. G. (1972) Cell Proliferation during Immunological Perturbation in Three Transplanted Tumours. *Br. J. Cancer*, **26**, 108.

KARLSON, A. G. & MANN, F. C. (1952) The Transmissible Venereal Tumor of Dogs: Observations of Forty Generations of Experimental Transfers. *Ann. N.Y. Acad. Sci.*, **54**, 1197.

KLIGERMAN, M. M., HEIDENREICH, W. F. & GREENE, S. (1962) Distribution of Tritiated Thymidine about a Capillary Sinusoid in a Transplanted Mouse Tumour. *Nature, Lond.*, **196**, 282.

LORD, B. I. (1963) Autoradiography with Tritium Labelled Cell Preparations: Some Physical Factors Affecting Image Production in Two Liquid Nuclear Emulsions. *J. photogr. Sci.*, **11**, 342.

MENDELSOHN, M. L. (1962) Chronic Infusion of Tritiated Thymidine into Mice with Tumors. *Science, N.Y.*, **135**, 213.

MENDELSOHN, M. L., DOHAN, F. C. & MOORE, H. A. (1960) Autoradiographic Analysis of Cell Proliferation in Spontaneous Breast Cancer of C3H Mouse. I. Typical Cell Cycle and Timing of DNA Synthesis. *J. natn. Cancer Inst.*, **25**, 477.

NOVINSKY, M. A. (1876) Zur Frage über die Impfung der Krebsigen Geschwülste. *Zentbl. med. Wiss.*, **14**, 790.

OWEN, L. N. & STEEL, G. G. (1969) The Growth and Cell Population Kinetics of Spontaneous Tumours in Domestic Animals. *Br. J. Cancer*, **23**, 493.

POWERS, R. D. (1968) Immunologic Properties of Canine Transmissible Venereal Sarcoma. *Am. J. vet. Res.*, **29**, 1637.

PRIER, J. E. & JOHNSON, J. H. (1964) Malignancy in a Canine Transmissible Venereal Tumor. *J. Am. vet. med. Ass.*, **145**, 1092.

QUASTLER, H. & SHERMAN, E. G. (1959) Cell Population Kinetics in the Intestinal Epithelium of the Mouse. *Expl. Cell Res.*, **17**, 420.

RUST, J. H. (1949) Transmissible Lymphosarcoma in the Dog. *J. Am. vet. med. Ass.*, **114**, 10.

STEEL, G. G. (1968) Cell Loss from Experimental Tumours. *Cell Tiss. Kinet.*, **1**, 193.

STEEL, G. G. (1972) The Cell Cycle in Tumours: An Examination of Data gained by the Technique of Labelled Mitoses. *Cell Tiss. Kinet.*, **5**, 87.

STEEL, G. G., ADAMS, K. & BARRETT, J. C. (1966) Analysis of the Cell Population Kinetics of Transplanted Tumours of Widely-differing Growth Rate. *Br. J. Cancer*, **20**, 784.

STEEL, G. G. & HANES, S. (1971) The Technique of Labelled Mitoses: Analysis by Automatic Curve-fitting. *Cell Tiss. Kinet.*, **4**, 93.

STICKER, A. (1906) Transplantables Rundzellensarkom des Hundes. Ein Beitrag zur Lehre der Krebsübertragbarkeit. *Krebsforsch.*, **4**, 227.

TANNOCK, I. F. (1968) The Relation Between Cell Proliferation and the Vascular System in a Transplanted Mouse Mammary Tumour. *Br. J. Cancer*, **22**, 258.

WRIGHT, D. H., PEEL, S., COOPER, E. H. & HUGHES, D. T. (1970) Transmissible Venereal Sarcoma of Dogs. A Histochemical and Chromosomal Analysis of Tumours in Uganda. *Rev. eur. Étud. clin. biol.*, **15**, 155.